# Pentraxin 3: a novel biomarker in pediatric central nervous system infections

**DOI:** 10.1186/s12887-024-05315-8

**Published:** 2025-01-06

**Authors:** Huda El-Kady, Mona Gamal Mostafa, Shaimaa Madkour

**Affiliations:** 1 Department of Pediatrics, Faculty of Medicine, Fayoum University, Fayoum, Egypt; 2 Department of Clinical and Chemical Pathology, Faculty of Medicine, Fayoum University, Fayoum, Egypt

**Keywords:** CNS infections, Pentraxin 3, Children, C-reactive protein, Meningitis, Aseptic meningoencephalitis, CSF

## Abstract

**Background:**

Pediatric CNS infections have been identified as a global health problem, associated with an increased death rate and fatal consequences. Pentraxin 3 (PTX3) is an acute-phase mediator that increases in body fluids and plasma throughout inflammation. Our study was designed to assess the diagnostic and prognostic value of cerebrospinal fluid (CSF) PTX3 levels in pediatric patients with different central nervous system (CNS) infections.

**Methods:**

We enrolled 100 children hospitalized at Fayoum University Children’s Hospital with suspected CNS infections fulfilling the case criteria for CNS infections. We recorded their medical history and examination data upon admission. The C-reactive protein (CRP) level, complete blood count (CBC), CSF PTX3 level, CSF analysis and culture, and blood culture were assessed in all patients at the time of admission.

**Results:**

Levels of PTX3 were significantly correlated with the duration of symptoms before admission, length of hospital stay, patient outcomes, CRP levels, CSF findings, and CSF cultures *(P value* < *0.001)*. Patients who needed mechanical ventilation or experienced adverse outcomes had greater levels of PTX3, which were more prevalent in those with a bacterial etiology *(P value* < *0.05).*

**Conclusion:**

PTX3 indicates disease severity and prognosis. PTX3 showed statistically significant sensitivity when discriminating between bacterial and aseptic CNS infections, as well as between bacterial CNS infections and controls. However, it has lower sensitivity and specificity than other CSF biomarkers, though it is higher than serum CRP.

## Background

In children, fever and central nervous system (CNS) symptoms are commonly attributed to CNS infections [1]. The incidence of CNS infections is greater in developing nations (726 cases per 100,000 persons) than in developed populations (11 per 100,000 people) [2, 3]. As a result, pediatric CNS infections are recognized as a global health problem, particularly in developing nations, where they are associated with a high death rate [4]. The documented incidence of bacterial meningitis in Egypt is high, ranging from 47 to 68% [5]. In Egypt, meningitis is considered an endemic disease [6].

In children, CNS infections can lead to fatal outcomes, neurological impacts, hearing loss, death, and other serious effects if left improperly treated [7]. The most prevalent form of CNS infection is meningitis [3, 8], although other forms include encephalitis and brain abscesses. Microbiological culture is considered the main method for diagnosis of CNS infection. However, the sensitivity of this procedure is limited, and the diagnosis could take longer [9, 10].

Furthermore, bacterial growth in microbiological cultures takes at least two days. Cultures are not available or take significantly longer in cases of viral meningitis. Multiplex real-time polymerase chain reaction (PCR) is a highly sensitive technique for detecting the most common viral and bacterial infections, shortening the time spent in the laboratory. Nevertheless, the limitation of this PCR-based molecular test is that it requires expensive equipment for molecular testing and expert specialists. In clinical settings, clinical symptoms, biochemical and cytological properties of cerebrospinal fluid (CSF), culture results, and neuroimaging results are typically used to make the first CNS infection diagnosis, including meningitis. The most common method for obtaining CSF to diagnose meningitis is a lumbar puncture [9, 11, 12].

Serum inflammatory biomarkers, such as interleukin-6, interleukin-10, and procalcitonin, have a reduced diagnostic ability for diagnosing bacterial meningitis and CNS infections [13].

The pentraxin superfamily comprises extremely conserved molecules with a similar structural motif known as the “pentraxin domain” [14–16]. The family’s prototypes, serum amyloid P component (SAP), and C-reactive protein (CRP), which was first identified for its capability to bind the C-polysaccharide of *Streptococcus pneumoniae*, make up the short pentraxin arm. The long N-terminal domain of long pentraxin 3, which was first discovered in the early nineties, is the prototype of the long pentraxin family and is a feature that distinguishes it from other proteins [14, 16].

Inflammatory mediators and tissue damage primarily generate multifunctional molecules such as CRP, SAP, and pentraxin 3 (PTX3). CRP is the most significant and is frequently measured to monitor various diseases. SAP may be a target for therapy and is involved in the development of amyloid. PTX3 is essential for regulating inflammation, tissue remodeling, and cancer, and it serves as an important mediator of innate immunity against certain bacterial, viral, and fungal pathogens [16].

The PTX3 molecule is stable in lab environments even after multiple freeze–thaw cycles [17, 18]. PTX3 is currently recognized as a marker for inflammatory, viral, and cardiovascular disorders [18–21].

Elevated PTX3 levels in plasma have been linked to infection severity, particularly in sepsis [22, 23]. A recent analysis revealed considerably higher plasma levels of PTX3 in severely ill patients and those with positive blood culture bacteremia than in healthy controls. Additionally, greater levels of PTX3 are related to the progress of septic shock and severe sepsis [23].

Although PTX3 is not regularly expressed in the brain, it may be generated centrally in response to proinflammatory signals [24]. The levels of PTX3 in CSF have been linked in certain studies to several CNS conditions, including multiple sclerosis, traumatic brain injury, subarachnoid hemorrhage, epilepsy, stroke, and neurodegenerative diseases (including Parkinson’s disease) [19]. Currently, there is little knowledge regarding the possible application of CSF PTX3 measurement in cases of CNS infections [18, 25].

Our goal was to assess CSF PTX3’s diagnostic and prognostic value in pediatric patients with various forms of CNS infection. Particularly, we intended to determine if PTX3 levels might be useful as a marker to differentiate aseptic meningoencephalitis from bacterial infections.

## Methods

### Study design

A case–control study was performed between February 2022 and February 2024 at Fayoum University Children’s Hospital.

### Sample size

G-Power© software 3.1.7 (Institute of Experimental Psychology, Heinrich Heine University, Dusseldorf, Germany) was used to identify the sample size. There will be 100 cases in the entire sample of participants. Based on findings from earlier studies, the power was set at 80%, the impact size was 0.407, and the two-sided (two-tailed) type I error was 0.05. To account for incomplete or missing data, the sample size was raised by 10%.

### Inclusion criteria

Based on the clinical evaluation, we selected 100 children, ranging in age from 1 month to 14 years, who fulfilled the clinical case definition for suspected CNS infection, as demonstrated in Table 1 [26, 27].

**Table 1:** Study clinical case definitions

Criterion	Definition
*Age*	1 month–14 years
*Fever*	≥ 38 °C throughout the first day of hospitalization
*Clinical features*	One or more of the following: • Neck stiffness • Altered/reduced consciousness ◦ V, P, or U on the AVPU score ◦ Glasgow Coma Score < 15 • Focal neurological symptoms/signs • Seizures ◦ Any seizure in children aged < 6 months or ≥ 6 years ◦ Any focal or prolonged seizure OR ≥ 2 brief generalized seizures in children aged 6 months to < 6 years • Bulging fontanelle if < 12 months of age • Irritable child if < 5 years of age • Headache • Prostration • Inability to drink or breastfeed, or to remain sitting in a child otherwise able to sit • Petechial or purpuric rash
*Laboratory* ***I****nvestigation*	The clinical team either performed or actively arranged a lumbar puncture during the assessment

*AVPU score* Alert, verbal, pain, unresponsive score

Enrollment in the study required meeting all four criteria (age, fever, clinical, and laboratory)

### Exclusion criteria

Included insufficient CSF samples (< 1.5 mL), patients with a history of fever before hospitalization and did not show fever during hospitalization, multisystem dysfunction, pediatric inherited metabolic disease, or other neurological diseases (such as cerebrovascular disease, subarachnoid hemorrhage, multiple sclerosis, brain tumors, traumatic brain injury, stroke, and neurodegenerative diseases).

### Patients

The patients were categorized into three groups according to the outcomes of the CSF analysis and the final diagnosis: patients with bacterial meningitis were assigned to Group A (40 patients), whereas patients with aseptic meningoencephalitis (mostly due to viral agents according to CSF analysis) were assigned to Group B (20 patients) and (40 patients) in the control group (Group C). The following are the group classification criteria: [28, 29]

Patients in the control group (Group C) were admitted with a suspected CNS infection; however, their CSF biochemistry and culture results were normal (protein < 40 mg/dL and white blood cell count under 10 × 10^6^ cells/L, respectively), and their clinical status improved without requiring specific treatment. Following these patients, they had many diagnoses other than CNS infections, such as complex febrile seizures, the first presentation of epileptic attacks precipitated by fever, acute adrenal crisis, and intoxications.

### Bacterial meningitis (Group A) inclusion criteria

Group A was further subdivided into 2 groups (with known and unknown pathogens).

#### Bacterial meningitis with known pathogens

Children with clinical manifestations suggesting bacterial meningitis, combined with one or more of the following criteria:

a CSF cell count ≥ 250 cells/mm with a dominance (≥ 60%) of polymorphonuclear neutrophils.
b Positive CSF culture.
c A positive blood culture, along with one or more CSF abnormalities indicated as follows: protein > 100 mg/dL and CSF glucose < 40 mg/dL.
d Bacteria were observed in the CSF Gram staining.

#### Bacterial meningitis with an unknown organism

The clinical criteria outlined above, along with the following CSF parameters, were taken into account: > 10 × 10^6^ cells/L with a dominance of polymorphonuclear neutrophils, low CSF glucose (< 40 mg/dL), and high protein content > 100 mg/dL. In addition, CSF samples may be turbid and have high opening pressure. This group of patients had negative microscopy, blood, and CSF cultures.

### Aseptic meningoencephalitis (Group B) inclusion criteria

The CSF aspect was clear with a normal opening pressure. Cerebrospinal leukocytes > 10 × 10^6^ cells/L with a mononuclear dominance with mildly elevated protein, mildly decreased glucose concentration, and no alternative diagnosis were considered (with serum laboratory findings indicating a viral infection and a complete clinical cure on antiviral therapies). Bacterial meningitis was excluded by microscopy, blood, and CSF cultures. Aseptic meningoencephalitis might be caused by various pathogens: [30, 31]

• Viruses (such as *Herpes, Varicella, Arbo-, and Entervirusus*).
• Bacteria (such as *Mycobacteria, Rickettsia, Mycoplasma, and Listeria Monocytogenes*).
• Fungi (such as *Cryptococci*).
• Parasitic (such as *Acanthamoeba* species).

### Methodology

Comprehensive clinical, demographic, biochemical, and bacteriological data from the blood and CSF were obtained. Short-term follow-up for neurological consequences was conducted, with results assessed in the month following discharge. Clinical outcomes were categorized at both discharge and follow-up as follows: complete recovery, neurological sequelae, or death.

A full history was taken, and a physical evaluation for focal neurological deficits was part of the follow-up hospital visit for neurological sequelae. Questions regarding neurological symptoms were also asked. Blood and CSF samples were collected on the day of admission.

### CSF analysis

CSF samples (3–4 mL) were collected by lumbar puncture. One milliliter of each sample was immediately stored at -80 °C for PTX3 measurement. The rest of the samples were subjected to Gram staining, Ziehl–Neelsen staining, glucose, chloride, lactate dehydrogenase (LDH), protein estimation (Cobas c 311 Roche Diagnostics GmbH Mannheim, Germany), and white blood cell counts (Sysmex XN-1000 SA-01, Japan, made in Germany) with Giemsa-stained slides for differential white blood cell counts. CSF was cultured on 5% sheep blood agar, chocolate agar, MacConkey, and Sabouraud agar plates (Oxoid, made onsite). Conventional microbiological methods, such as colony morphology, Gram stain appearance, growth requirements, and confirmatory biochemical/serological testing, were used to identify bacterial isolates from blood and CSF cultures. Additionally, if physicians suspect a cryptococcal infection, they could ask for India ink staining.

### Measurement of CSF pentraxin 3 Level

CSF samples were stored at -80 °C for PTX3 measurement. CSF samples were centrifuged at 2000–3000 rpm for 10 min prior to analysis. A human PTX3 double-antibody sandwich enzyme-linked immunosorbent assay (ELISA) kit (Bioassay Technology Lab, Cat. No. E1938Hu, China) was used to test the samples for PTX3. Using an automatic plate reader, the optical densities (ODs) of the samples and standards were measured at a wavelength of 450 nm. The concentration was determined by converting the optical density readings against a standard curve made with standard samples provided by the kit.

### Statistical analysis

The data were analyzed with the Statistical Package for Social Science (SPSS) software version 22 on Windows 7 (SPSS Inc., Chicago, IL, USA). Simple descriptive analysis of qualitative data utilizing numbers and percentages, arithmetic means to measure central tendency, and standard deviations to measure dispersion for quantitative parametric data. Quantitative data were analyzed using the Student t-test, Mann–Whitney U tests, one-way ANOVA-F test, and Kruskal–Wallis H test. A post-hoc analysis was conducted to compare each 2 groups. The chi-square test is used to analyze qualitative data. To determine the association between quantitative variables, the bivariate Spearman correlation test was used. The ROC curve “Receiver Operating Characteristic” was utilized for the sensitivity and specificity tests. *P-*values < 0.05 were considered statistically significant.

## Results

There were one hundred patients in the current study. They were categorized into three groups: *40* patients from Group *A* had *bacterial meningitis*, *20* patients from Group* B* had *aseptic meningoencephalitis*, and *40* patients from Group *C* composed *the control group*.

There was no statistically significant variation in age or sex among the cases and controls, as indicated in Table 2.

**Table 2:** Comparisons of demographic characteristics across the different study groups

**Variables**	**Group A (*N*=40)**		**Group B** **(*N*=20)**		**Control (*N*=40)**		***P*-value**
**Age (months)**							
** Mean ±SD**	8.9±13.4		12.3±11.9		10.7±9.1		0.55#
**Sex**							
** Male**	22	55%	14	70%	16	40%	0.08$
** Female**	18	45%	6	30%	24	60%	

*SD* Standard deviation

#Kruskal-Wallis H test

$Chi-square test

**Fig. 1:**
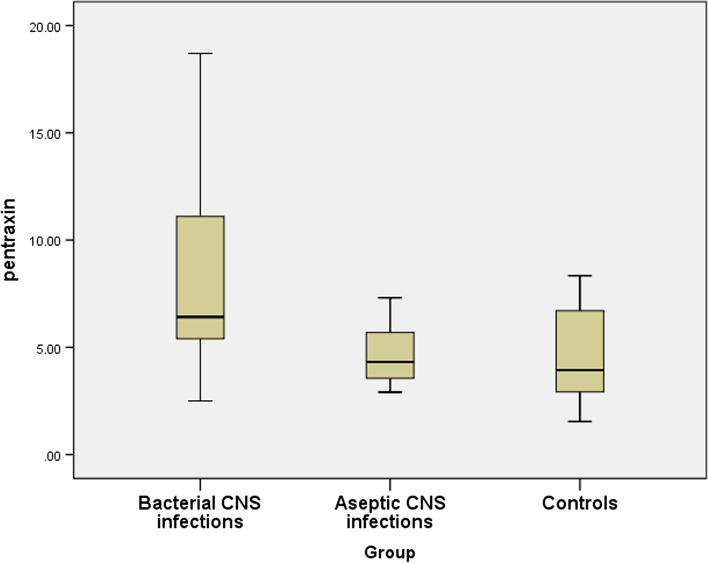
Comparisons of pentraxin-3 levels among the different study groups

PTX3 levels were significantly greater in patients with bacterial meningitis *(p-value* < *0.001)*, as presented in Table 3 and Fig. 1.

**Table 3:** Comparisons of pentraxin-3 levels in different study groups

Variables	Pentraxin-3 level (ng/ml)		*P-*value
	**Mean**	**SD**	
**Group A**	8.3	4.4	< 0.001*a,b 0.38c
**Group B**	4.7	1.4	
**Control**	4.6	2.01	

Kruskal–Wallis H test “between three groups” *p-*value < 0.001*

Mann–Whitney U tests (Post-hoc between each two groups)”

^a^between groups A, & B

^b^between groups A, & control

^c^between groups B, and control

Table 4 shows that most patients with bacterial meningitis were hospitalized in the Pediatric Intensive Care Unit (PICU) and experienced high-grade fever and vomiting. In addition, they experienced a significantly prolonged duration of symptoms before admission, along with an extended hospital stay. Additionally, their examination also revealed abnormalities, including hypertonia and hyperreflexia. Finally, these patients had a greater percentage of deaths and unfavorable outcomes (sequelae in the form of developmental delay, motor deficits, and epilepsy that necessitate long-term antiepileptic drug therapy), with a *p-value* < *0.05*. However, cases of aseptic meningoencephalitis had a significantly greater percentage of seizures.

**Table 4:** Comparison of clinical assessment measures across different study groups

**Variables (*n* = 100)**	**Total (*N* = 100)**	**Study groups**			***p*-value**
		**Group A** (*N* = 40)	**Group B** (*N* = 20)	**Control** (*N* = 40)	
***Site of admission***					
** PICU**	52 (52%)	*28(70%)	4(20%)	20(50%)	0.004*
** Ward**	48 (48%)	12(30%)	16(80%)	20(50%)	
***Clinical features***					
** Duration of symptoms before admission (Days) (Mean ± SD)**		*4.9 ± 2.3	3 ± 1.6	2.7 ± 1.7	<0.001*
** Hospital stay duration (Days) (Mean ± SD)**		*20.6 ± 8.3	11.8 ± 10.1	2.4 ± 1.2	<0.001*
** Preceding respiratory symptoms**	6(6%)	2(5%)	2(10%)	2(5%)	0.70
**Fever**					
Low grade	60 (60%)	16(40%)	*14(70%)	30(75%)	<0.001*
High grade	40 (40%)	24(60%)	6(30%)	10(25%)	
**Irritability**	6(6%)	2(5%)	2(10%)	2(5%)	0.70
**Vomiting**	10 (10%)	*8(20%)	2(10%)	–-	0.01*
**DCL**	24 (24%)	10(25%)	2(10%)	12(30%)	0.23
**Seizures**	62 (62%)	28(70%)	*20(100%)	14(35%)	<0.001*
**Neck rigidity**	2 (2%)	2(5%)	–-	–-	0.22
**Normal tone and reflexes**	76 (76%)	20(50%)	16(80%)	40(100%)	<0.001*
**Hypertonia & hyperreflexia**	24 (24%)	*20(50%)	4(20%)	–-	
***Management***					
** No mechanical ventilation**	82 (82%)	26(65%)	20(100%)	36(90%)	0.001*
** Mechanical ventilation**	18 (18%)	*14(35%)	0(0%)	4(10%)	
***Outcome***					
** Normal**	74 (74%)	16(40%)	18(90%)	40(100%)	<0.001*
** Sequelae**	16 (16%)	16(40%)	0(0%)	0(0%)	
** Died**	10 (10%)	*8(20%)	2(10%)	0(0%)	

Chi-square test

*PICU* pediatric intensive care unit, *SD* standard deviation, *DCL* disturbed conscious level, *:significant

Hemoglobin levels were significantly lower (*p-value* < *0.05*), whereas platelet counts were considerably elevated in cases of aseptic meningoencephalitis. The serum levels of CRP were significantly greater in bacterial cases than in aseptic and control cases. In the CSF examination, patients diagnosed with bacterial meningitis presented considerably greater total leucocyte counts (TLC), neutrophil counts, protein, LDH, and chloride levels, as well as significantly lower levels of glucose and lymphocytes (*p-value* < *0.001*). These data are explained in Table 5.

**Table 5:** Comparison of laboratory investigations in different study groups

**Variables** (*n* = 100)	**Study groups**			***p*-value**
	**Group A** (*N* = 40)	**Group B** (*N* = 20)	**Control** (*N* = 40)	
***CBC***				
Hemoglobin (g/dl)	9.5 ± 1.2	8.4±0.67*	10.4 ± 1.9	0.02*a 0.01*b < 0.001*c
TLC (10^3^/mm^3^)	11.5 ± 5.6	10.4 ± 2.4	10.9 ± 3.2	0.84
PLT (10^3^/mm^3^)	331.2 ± 166.1	372 ± 68.5	*260.4 ± 93.9	0.70a 0.03*b 0.004*c
*** CRP(mg/L)***	*46.8 ± 32.9	**21.1 ± 14.5**	**16.8 ± 16.1**	< 0.001*a,c 0.99c
***CSF analysis***				
TLC (/mm^3^)	*7571.5 ± 1867.2	87.1 ± 68.9	5.5 ± 6.5	0.06a 0.01*b 0.99c,
Neutrophils %	81.5 ± 13.6	14.7 ± 12.4	11.9 ± 29.7	< 0.001*a,b 0.99c
Lymphocytes %	*18.5±13.8	84.7 ± 12.8	88.7 ± 29.6	< 0.001*a,b 0.56c
Glucose (mgldL)	*16.1 ± 32.02	45.1 ± 13.7	75.5 ± 36.7	0.003*a < 0.001*b 0.002*c
Protein (mg/dL)	376.8 ± 127.6	66.8 ± 13.8	21.5 ± 9.1	< 0.001*a,b **0.13c**
LDH (IU/L)	*1322.4 ± 2223.03	36.9 ± 12.7	18.7 ± 22.2	0.003*a 0.001*b 0.99c
Chloride (mmol/L)	*136.4 ± 55.03	103.03 ± 7.4	112.2 ± 9.4	0.003*a 0.008*b 0.99c

Kruskal–Wallis H test “between three groups” *p-*value < 0.001*- Mann–Whitney U tests (Post-hoc between each two groups)”

*CBC* complete blood count, *TLC* total leucocyte count, *LDH* lactate dehydrogenase, *CRP* C-reactive protein, *CSF* cerebrospinal fluid

^a^between groups A, & B

^b^between groups A, & control

^c^between groups B, and control

With respect to the CSF culture data, bacterial meningitis was present in 65% of the samples with growth. Pseudomonas growth was observed in 46.2% of the cases. The most common resistance pattern was multidrug-resistant bacteria (MDR), which accounted for 46.2%. In terms of the blood culture results, 5% of the samples presented an increase in meningitis caused by bacteria.* Methicillin-resistant Staphylococcus (MRSA)* accounted for all the growth in blood cultures, as delineated in Table 6.

**Table 6:** Comparison of CSF and culture results between the study groups

**Variables** (*n* = 100)	**Study groups**			***p*-value**
	**Group A** (*N* = 40)	**Group B** (*N* = 20)	**Control** (*N* = 40)	
***CSF culture***				
No growth	14(35%)	20(100%)	40(100%)	< 0.001*
Growth	*26(65%)	–-	–-	
**Type of growth (*n* = 26)**				
* Klebsiella*	8(30.8%)	–-	–-	–-
* Pseudomonas*	12(46.2%)	–-	–-	
* Gram negative bacilli*	6(23.1%)	–-	–-	
**Type of resistance**				
MDR	12(46.2%)	–-	–-	––
***Blood culture***				
No growth	38(95%)	20(100%)	40(100%)	0.22
Growth	2(5%)	–-	–-	
**Type of growth (*n* = 2)**				
*MRSA*	2(100%)	–-	–-	

Chi-square test

*CSF* cerebrospinal fluid, *MDR* multidrug-resistant bacteria, *MRSA* methicillin-resistant Staphylococcus aureus, *:significant

The PTX3 levels were considerably greater in patients who required mechanical ventilation and had sequelae (*p-value* < *0.05*), as displayed in Table 7.

**Table 7:** Comparison of the levels of Pentraxin-3 according to different clinical assessment measures across the different study groups

**Variables **(*n* = 100)	**Pentraxin-3 level (ng/ml)**		***p*-value**
	**Mean**	**SD**	
***Site of admission***			
PICU	6	3.8	0.33
Ward	6.2	3.4	
***Examination***			
Normal	5.9	3.8	0.65
Hypertonia & hyperreflexia	6.4	2.7	
***Management***			
No mechanical ventilation	5.8	3.7	0.01*
Mechanical ventilation	7.3*	2.8	
***Outcome***			
Normal	5.7	3.5	0.99a 0.01*b 0.07c
Sequaelae	5.9	0.61	
Death	*9.2	5.5	

Kruskal–Wallis H test “between three groups” *p-*value < 0.001*- Mann–Whitney U tests (Post-hoc between each two groups)”

*PICU* Pediatric intensive care unit, *SD* Standard deviation

^a^between groups A, & B

^b^between groups A, & control

^c^between groups B, and control

As shown in Table 8, positive growth in the CSF culture was related to a statistically significant increase in PTX3 levels (*p-value* < *0.001*), independent of the organism type. Nevertheless, there was no statistically significant difference in the PTX3 level among the findings of blood cultures.

**Table 8:** Comparisons of pentraxin-3 levels in different blood and CSF culture findings in the study group

Culture results	Pentraxin -3 level (ng/ml)		*P-*value
	**Mean**	**SD**	
***Blood culture***			
No growth	6.1	3.6	0.81
Growth	5.5	0	
***CSF culture***			
No growth	4.9	1.7	< 0.001*
Growth	9.5	5.1	
**Type of CSF growth (*n* = 26)**			
* Klebsiella*	10.8	6.4	0.53
* Pseudomonas*	9.6	4.8	
* Gram negative bacilli*	7.7	3.8	

Kruskal–Wallis H test “between three groups” *p-*value < 0.001*- Mann–Whitney U tests (Post-hoc between each two groups”)

*SD* Standard deviation, *CSF* Cerebrospinal fluid

PTX3 levels were elevated (*p-value* < *0.05*) in those with higher CRP levels, longer hospital stays, and older ages. In the CSF study, there was a significant association (*p-value* < *0.05*) between an increase in the pentraxin-3 level and increases in TLC, the neutrophil percentage, protein, LDH, and a decrease in glucose and lymphocyte percentages, as indicated in Table 9.

**Table 9:** Correlations between the pentraxin-3 level and clinical data in the study group

Variables	Pentatraxin-3 level	
	**R**	***P-*value**
Age (months)	**-0.29**	0.003*
Duration of symptoms before admission (days)	0.19	0.06
Hospital stay (days)	**0.34**	0.001*
**CBC**		
HB (g/dl)	0.12	0.22
TLC (10^3^/mm^3^)	-0.08	0.45
PLT (10^3^/mm^3^)	-0.12	0.22
CRP (mg/L)	**0.42**	< 0.001*
**CSF analysis**		
TLC (/mm^3^)	**0.50**	< 0.001*
Neutrophils %	**0.38**	< 0.001*
Lymphocytes %	**-0.37**	< 0.001*
Glucose (mg/dL)	**-0.41**	< 0.001*
Protein (mg/dL)	**0.46**	< 0.001*
LDH (IU/L)	**0.43**	< 0.001*
Chloride (mmol/L)	0.12	0.24

Bivariate Spearman correlation test

*CBC* complete blood count, *TLC* total leucocyte count, *CRP* C-reactive protein, *LDH* lactate dehydrogenase, *CSF* cerebrospinal fluid, *:significant

**Fig. 2:**
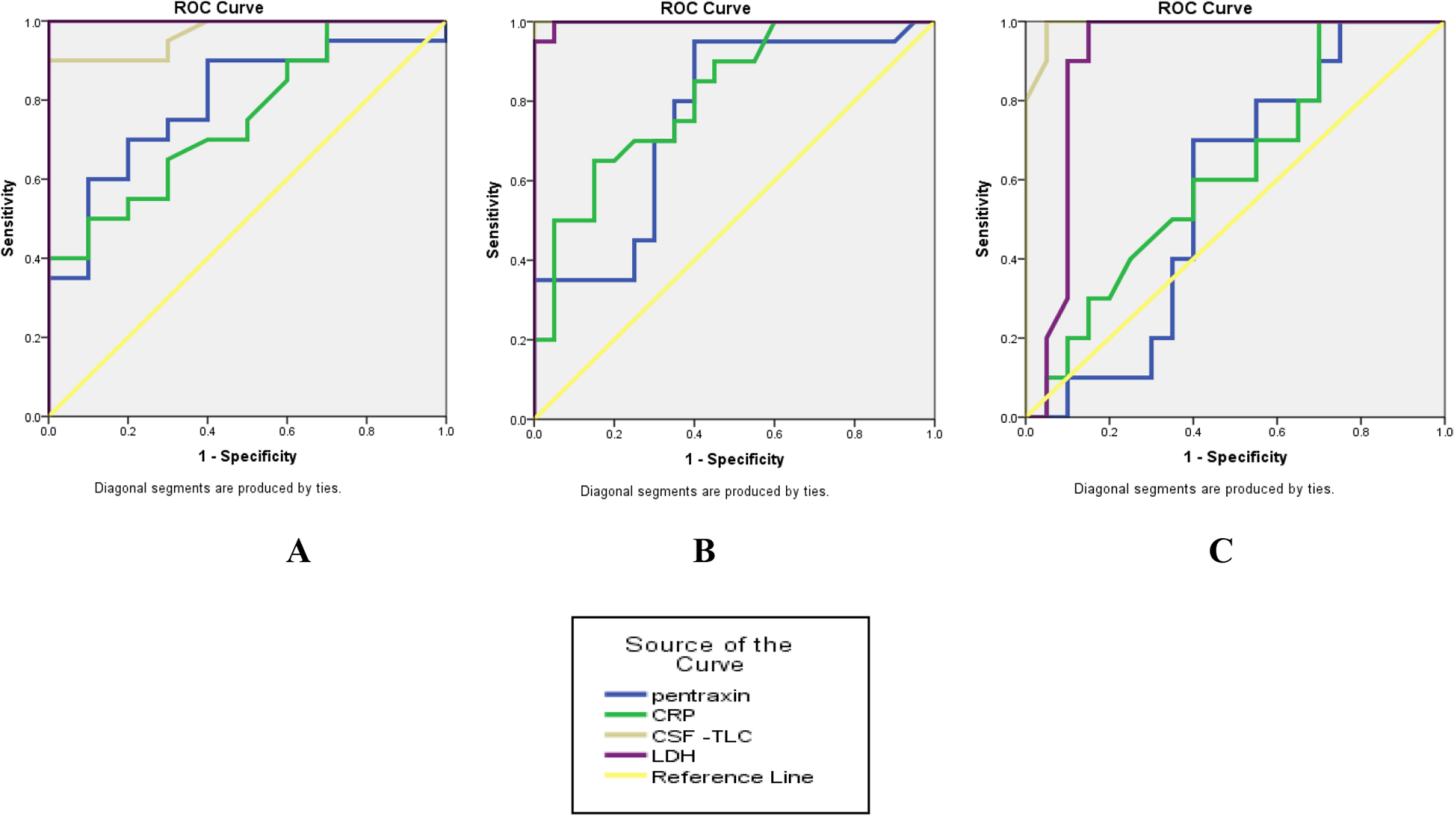
ROC curve for PTX 3, CRP, CSF-TLC, and LDH in: **A** Diagnosis of bacterial versus aseptic CNS infection. **B** Diagnosis of bacterial CNS infection versus controls. **C** Diagnosis of aseptic CSF infection versus control*.* ROC: receiver operating characteristic. CSF-TLC: cerebrospinal fluid total leucocyte count. LDH: lactate dehydrogenase. CRP: C-reactive protein

The PTX3 and CRP levels demonstrated statistically significant sensitivity in diagnosing bacterial vs. aseptic CNS infections. However, they were unable to distinguish aseptic CNS infection from the control group. At a cut-off of 5.42 ng/ml, which differentiates bacterial from aseptic CNS infections, 10/40 (25%) bacterial CNS infections were false negatives and 6/20 (30%) aseptic CNS infections were false positives. This resulted in a sensitivity of 75%, specificity of 70%, PPV of 83.3%, NPV of 58.3%, and an AUC of 79.5%. CSF-TLC and LDH levels had a statistically significant sensitivity in diagnosing bacterial versus aseptic CNS infection and in each group of cases versus controls (*p-value* < *0.001*). These findings are explained in Table 10 and Fig. 2.

**Table 10:** Sensitivity and specificity of the pentraxin-3 level in the diagnosis of patients

Variable	Sensitivity	Specificity	PPV	NPV	AUC	Cut off point	*P-*value	(95% CI)
**A) Bacterial versus aseptic CNS infection**								
Pentraxin-3 **(ng/ml)**	75%	70%	83.3%	58.3%	79.5%	5.42	< 0.001*	(0.679–0.911)
CRP (mg/L)	75%	50%	75%	50%	75.3%	21	< 0.001*	(0.629–0.876)
CSF-TLC (/mm^3^)	95%	70%	86.4%	87.5%	96.7%	111	< 0.001*	(0.93–1)
LDH (IU/L)	95%	100%	100%	90.9%	100%	207.5	< 0.001*	(1–1)
**B) Bacterial CNS infection versus control**								
Pentraxin-3 **(ng/ml)**	80%	65%	69.6%	76.5%	75.9%	5.28	< 0.001*	(0.651–0.867)
CRP (mg/L)	85%	60%	68%	80%	81.4%	19.2	< 0.001*	(0.722–0.905)
CSF-TLC (/mm^3^)	100%	100%	100%	100%	100%	69.5	< 0.001*	(1–1)
LDH (IU/L)	100%	95.5%	95.2%	100%	99.8%	70	< 0.001*	(0.992–1)
**C) Aseptic CNS infection versus control**								
Pentraxin-3 **(ng/ml)**	70%	60%	46.7%	80%	57%	4.16	0.38	(0.426–0.714)
CRP (mg/L)	70%	45%	38.9%	75%	61.8%	9.5	0.14	(0.472–0.763)
CSF-TLC (/mm^3^)	100%	95.5%	90.9%	100%	99.3%	18.5	< 0.001*	(0.979–1)
LDH (IU/L)	100%	85%	75%	94.4%	90.8%	25.5	< 0.001*	(0.824–0.991)

ROC curve “Receiver Operating Characteristic” test

*AUC* area under the curve, *NPP* negative predictive value, *PPV* positive predictive value, *CNS* central nervous system, *CSF-TLC* cerebrospinal fluid total leucocyte count, *LDH* lactate dehydrogenase, *CRP* C-reactive protein, *CI* confidence intervals, *:significant

## Discussion

The current research assessed the levels of CSF PTX 3 in different CNS infections and compared the results with those of other blood and CSF biomarkers. Only a limited number of studies have explored the connection between PTX3 and CNS infections. Sprong et al. [21] concluded that subjects with meningococcal meningitis-related septic shock had considerably greater plasma concentrations of PTX3 than did those without shock; nonetheless, intrathecal PTX3 levels were examined in only 2 studies (Zatta et al. [18] and Thomsen et al. [25]). In addition, as far as we know, this is the first study conducted within a pediatric age group.

This study comprised 100 patients suspected of having CNS infections, according to the study’s case definition. These findings revealed that PTX 3 was sensitive in differentiating bacterial meningitis from aseptic meningoencephalitis and control patients. However, it could not differentiate between aseptic patients and controls.

Our findings further confirmed previous findings that male children are more prone to neurological infections, which is consistent with the findings of Marinho et al. [3].

The clinical symptoms and signs of viral and bacterial infections are similar, making it critical to examine both clinical and laboratory data for an appropriate diagnosis. The most common initial symptom observed in our patients was fever, affecting all patients, followed by seizures (62%), altered sensorium (24%), vomiting (10%), and irritability (6%). Thus, fever, seizures, and altered sensorium, which are regarded as a triad of symptoms in acute CNS infections, were evident in the majority of cases [1]. This is in line with a previous study by Vasavada et al. [1], which found that seizures were more frequent in cases of viral infections, whereas vomiting was more common among patients with bacterial infections. Unlike Marinho et al.[3] and other studies [32], which attributed altered consciousness to viral etiologies. None of our patients complained of photophobia, headache, bulging fontanels, or a meningitic rash.

In our research, 10% of our patients died (the majority were diagnosed with bacterial meningitis), and 16% had neurological sequelae. This rate was higher than that reported by Ai et al. [33], who found a mortality rate of 0.8% in patients with only viral etiologies, and Turner et al. [34], who reported a mortality rate of 2.5% in patients with various etiologies. However, our outcomes were lower than those of another [35] study, which reported 13% deaths and 27% unfavorable outcomes (neurological sequelae). The levels of CSF PTX3 were notably higher in patients who experienced poor outcomes (deaths or unfavorable sequelae). Thomsen et al. [25], however, reported findings that contradicted ours.

Our results align with those reported by Mount et al. [36] and another study [3], both of which revealed the presence of pleocytosis with predominant neutrophils, high protein levels, and low glucose levels, indicating bacterial infection in the CSF. On the other hand, viral infections have unique presentations, including normal glucose levels, slight protein increases, and mononuclear pleocytosis.

Interestingly, our research revealed statistically significant differences in CSF biochemical and cellular outcomes between viral and bacterial infections, suggesting changes in glucose, protein levels, and cellularity. These findings align with those presented by the research of Nazir et al.’s research [37] and Marinho et al. [3], which highlighted the importance of CSF profile changes as a critical marker for distinguishing bacterial infections from viral infections.

Our findings were supported by Kazancioglu et al. [38], who reported that LDH in cerebrospinal fluid (CSF) was useful for predicting bacterial meningitis.

Our findings of a relationship between PTX3 and CSF polymorphonuclear cells (neutrophils) align with earlier research by Jaillon et al. [24], which revealed the storage of PTX3 in neutrophil granules. Additionally, the results of Thomsen et al. [25] were consistent with our findings.

In our study, the most common bacterial organism was Pseudomonas (46.2% of CSF growth), followed by *Klebsiella* (30.8%) and *Gram-negative bacilli* (23.1%). These results contradicted the findings of Farag et al. [5], who listed *N. meningitides*, *S. pneumoniae,* and *H. influenzae* as the most common causative organisms. Additionally, our results did not align with those of a study by Alnomasy et al. [39], which reported *meningococcal meningitis* as the most common bacterial infection. Several investigations, contrary to our findings, have revealed that viral meningitis is more common than bacterial meningitis. For instance, Dawod et al. (2019) documented that viral meningitis accounted for 87.1% of CNS infections [6]. Similarly, Agueda et al. (2013) found that viral infection accounts for 52.9% of meningitis cases [40].

The diversity of results could be explained by many factors, such as different geographic and demographic distributions, techniques used for pathogen detection, and the immunization programs applied in each region. The absence of *Haemophilus influenzae type B* and *meningococcal meningitis* cases in our analysis was predicted by routine immunization. PCV13 (pneumococcal conjugate vaccine 13) is predicted to have a similar effect on pneumococcal cases [41, 42].

In agreement with our results, Zatta et al. [18] and Thomsen et al. [25] mentioned that patients with culture-confirmed bacterial meningitis had considerably greater CSF PTX3 levels than those with negative cultures unrelated to isolated pathogens.

We discovered that PTX3 levels were significantly higher in the CSF of patients hospitalized with culture-confirmed bacterial meningitis compared to those admitted with aseptic meningoencephalitis, as well as to the control group of patients without CNS infection. Our findings support the notion that PTX3 can serve as a biomarker to differentiate bacterial meningitis from viral illnesses, making it a valuable clinical decision tool for excluding bacterial meningitis.

Compared with the results of other biomarkers, the NPV and PPV for CSF PTX3 were comparable to those of known biomarkers, such as CSF cell count, CSF LDH level, and CSF protein concentration.

In this work, PTX3 demonstrated a statistically significant ability to differentiate between bacterial and aseptic infections, with 75% sensitivity, 70% specificity, 83.3% positive predictive value (PPV), 58.3% negative predictive value (NPV), and an area under the curve (AUC) of 79.5% at a cut-off point of 5.42 ng/mL. These results were lower than those of other study parameters, such as CSF-TLC and LDH. The sensitivity and specificity of PTX3 in our research were comparable to those reported by Zatta et al. [18], who reported values of 71.4% and 91.4%, respectively, at a higher cut-off point of 9.6 ng/ml.

When comparing the diagnostic capabilities of CSF PTX3, CSF LDH, and CSF TLC, CSF PTX3 demonstrated lower sensitivity, specificity, PPV, and NPV in discriminating bacterial meningitis from aseptic meningoencephalitis than CSF LDH and CSF TLC, but it performed better than plasma CRP. Alternatively, Thomsen et al. [25] reported that CSF PTX3 has a better ability to differentiate them than CSF TLC does. Zatta et al. [18] reported that CSF PTX3 was better than plasma CRP at distinguishing them, but had lower ability than CSF TLC.

As mentioned previously, the diagnostic ability of CSF PTX3 for aseptic meningoencephalitis in controls was lower than that of CSF TLC and LDH, but greater than that of plasma CRP. However, Thomsen et al. [25] reported higher sensitivity and specificity, as well as better diagnostic value than CSF-TLC. This may be attributed to differences in sample size, as well as demographic and technical factors.

This study has several limitations. First, few participants were available because we conducted a single-center study in a single Egyptian city using the same laboratory; thus, the findings may not be applicable to the entire population. Second, CSF data were prospectively gathered, and samples were stored for later use. Throughout the study period, some samples were kept frozen. We are uncertain of how much this may have affected the outcomes and whether they would vary if fresh samples had been measured. Consequently, to generalize the results, multicenter trials using fresh samples are recommended. We suggest measuring it in different settings and via various methods. Third, plasma levels of PTX 3 were not assessed, and we recommend comparing CSF and plasma PTX 3 levels. Fourth, serial measurement is recommended for better assessment of severity and prognosis. Fifth, while we were able to detect bacterial pathogens using standard culture techniques, our limited resources prevented us from using serological, PCR, or special culture techniques to identify several other fatal species, including viruses, fungi, and atypical bacterial organisms such as Mycoplasma. As a result, viral cases are assessed based on CSF analysis, which is not an accurate method. Sixth, this is the first study on children with CNS infections, and only two studies have assessed adult intrathecal PTX3 in CNS infection. Lastly, owing to limited resources, the authors were unable to evaluate the correlation between PTX3 levels and other inflammatory biomarkers, such as procalcitonin, lactate, and neopterin.

## Conclusion

In this study, we investigated the role of CSF PTX 3 in the diagnosis of CNS infections and its ability to differentiate between various etiologies of CNS infections. PTX3 had cut-off values with comparable sensitivity, specificity, PPV, and NPV to those of other CSF biomarkers. It is slightly lower than the CSF LDH and CSF TLC but higher than the plasma CRP. PTX 3 showed statistically significant sensitivity in identifying bacterial vs. aseptic CNS infections (sensitivity 75%, specificity 70%, PPV 83.3%, NPV 58.3%, AUC 79.5% at the cut-off point of 5.42), as well as bacterial CNS infections compared with controls. However, it was unable to distinguish between aseptic CNS infection and the control group. More studies should be performed to validate our findings and illustrate the possible diagnostic and predictive significance of PTX3 assessment in the CSF. Combining PTX3 with other serum and CSF biomarkers could improve the practical applicability of predicting the type and severity of CNS infections.

## Data Availability

The datasets used in the current study are available from the corresponding author upon reasonable request.

## Abbreviations

AUC: Area under the curve
AVPU score: Alert, verbal, pain, unresponsive score
CBC: Complete blood count
CNS: Central nervous system
CRP: C-reactive protein
CSF: Cerebrospinal fluid
CSF-TLC: Cerebrospinal fluid total leucocyte count
DCL: Disturbed conscious level
LDH: Lactate dehydrogenase
MDR: Multidrug-resistant bacteria
MRSA: Methicillin-resistant Staphylococcus aureus
NPV: Negative predictive value
PCR: Polymerase chain reaction
PCV-13: Pneumococcal conjugate vaccine 13
Pentraxin 3: PTX3
PICU: Pediatric intensive care unit
PPV: Positive predictive value
ROC: Receiver operating characteristic
SAP: Serum amyloid P component
SD: Standard deviation
SPSS: Statistical Package of Social Science
TB: Tuberculosis
TLC: Total leucocyte count
WHO: World Health Organization
